# Causal Associations Between Serum Bilirubin Levels and Decreased Stroke Risk: A Two-Sample Mendelian Randomization Study

**DOI:** 10.1161/ATVBAHA.119.313055

**Published:** 2019-12-05

**Authors:** Yoonjeong Choi, Sun Ju Lee, Wes Spiller, Keum Ji Jung, Ji-Young Lee, Heejin Kimm, Joung Hwan Back, Sunmi Lee, Sun Ha Jee

**Affiliations:** 1 From the Department of Public Health, Graduate School, Yonsei University, Seoul, Korea (Y.C., S.H.J.); 2 Department of Epidemiology, Institute for Health Promotion, Graduate School of Public Health, Yonsei University, Seoul, Korea (Y.C., S.J.L., K.J.J., J.-Y.L., H.K., S.H.J.); 3 Population Health Science Institute, University of Bristol, Barley House, Oakfield Grove, United Kingdom (W.S.); 4 Health Insurance Policy Research Institute, National Health Insurance Service, Wonju, Korea (S.J.L., J.H.B., S.L.).

**Keywords:** bilirubin, cardiovascular diseases, causality, single nucleotide polymorphism, stroke

## Abstract

**Objective:**

A number of epidemiological studies have reported that decreased serum bilirubin, an endogenous antioxidant, is associated with cardiovascular disease. However, previous Mendelian randomization analyses conducted using a single sample have shown no evidence of association.

**Approach and Results:**

A 2-sample summary Mendelian randomization study was performed by obtaining exposure and outcome data from separate nonoverlapping samples. We utilized data from the KoGES (Korean Genome and Epidemiology Study; n=25 406) and KCPS-II (Korean Cancer Prevention Study-II; n=14 541) biobank for serum bilirubin and stroke, respectively. Using KoGES, a total of 1784 single nucleotide polymorphisms associated with serum bilirubin levels were discovered using a genome-wide significance threshold (*P*<5×10^−8^), of which 10 single nucleotide polymorphisms were identified as independent (*R*^2^<0.005) and adopted as genetic instruments. From KCPS-II, total and ischemic stroke cases were identified (n=1489 and n=686), with 12 366 acting as controls. Various 2-sample summary Mendelian randomization methods were employed, with Mendelian randomization estimates showing an inverse causal association between serum bilirubin levels and total stroke risk (odds ratio, 0.481 [95% CI, 0.234–0.988]; *P*=0.046). This association increased in magnitude when restricting the analysis to ischemic stroke cases (odds ratio, 0.302 [95% CI, 0.105–0.868]; *P*=0.026).

**Conclusions:**

Our findings provide evidence of significant causal relationship between high levels of bilirubin and decreased stroke risk in Korean population in agreement with observational approaches. This highlights the potential for bilirubin to serve as a therapeutic target for oxidative stress-related diseases such as stroke and suggests that previous findings were not a consequence of unmeasured confounding.

## Abstract 2

Supplemental Digital Content is available in the text.

**Highlights**

- This Mendelian Randomization study revealed that serum bilirubin level is causally associated with decreased stroke risk.
- A magnitude of association became stronger when the outcome is limited to ischemic stroke.
- Bilirubin may be suggested as a potential therapeutic target to the oxidative stress-related diseases, such as stroke.

Serum circulating bilirubin is widely known as a secondary degradation product of heme, which is released from the breakdown of aging red blood cells.^1^ Under physiological conditions, unconjugated plasma bilirubin concentrations in humans typically range from ≈5 to 17 µmol/L.^2^ Abnormally high concentrations of bilirubin are considered toxic and are linked to clinical symptoms of kernicterus^3^ or neurological dysfunction due to the deposition of bilirubin in the brain.^2^ However, mild to moderately elevated serum bilirubin concentrations can serve as a potent endogenous antioxidant, through the process of bilirubin oxidizing itself to biliverdin.^1,4^ This is supported by studies finding concentrations of total serum bilirubin <7 µmol/L to be a risk factor for oxidative stress-related diseases in humans, such as cardiovascular disease, diabetes mellitus, metabolic syndrome, certain cancers, and autoimmune diseases.^4^

Stroke is one such form of oxidative stress-related disease^5–7^ and has been widely reported to be inversely associated with serum bilirubin levels in both Western^8,9^ and Asian populations.^10,11^ However, such findings could result from unmeasured confounding factors, and as a consequence may not represent a true causal relationship. One strategy to correct for bias due to unmeasured confounding is Mendelian randomization (MR), which uses genetic variants associated with an exposure of interest to estimate the effect of genetically predicted serum bilirubin on stroke risk. This has the potential to provide unbiased causal association estimates, provided genetic variants are associated with the exposure of interest (IV1), are independent of confounders of the exposure and outcome (IV2), and are independent of the outcome after conditioning on the exposure (IV3). Associations violating assumptions IV2 and IV3 are generally referred to as horizontal pleiotropic associations.

In previous work, we performed a single sample MR analysis within a Korean population (n=5599), with estimates suggesting a possible inverse association between serum bilirubin and total stroke risk (hazard ratio, 0.63 [95% CI, 0.30–1.33]; *P*=0.229).^12^ However, the sample size proved insufficient to provide sufficient statistical power to identify a causal relationship between serum bilirubin and total stroke risk at conventional significance thresholds.

Our previous study provided motivation to conduct a 2-sample summary MR study, using data from the KoGES (Korean Genome and Epidemiology Study; n=25 406) and KCPS-II (Korean Cancer Prevention Study-II; n=13 855). By using 2 nonoverlapping samples, it has been possible to increase the sample size by a factor of 7 in comparison to our previous study. Further, adopting the 2-sample MR framework allows for several sensitivity analyses to be performed, critically examining the underlying assumptions of the MR approach (IV1-IV3). In this present study, we re-examine whether elevated serum bilirubin levels are causally associated with decreased stroke risk using 2-sample summary MR, interpreting the findings within the context of previous observational and MR studies.

## Materials and Methods

The data that support the findings of this study are available from the corresponding author on reasonable request.

### Selection of the Genetic Instrumental Variables

Candidate genetic instruments for total serum bilirubin levels were selected from KoGES. KoGES is a community-based cohort study, collecting data on ≈210 000 individuals from 2005 to 2014. From the KoGES cohort, 33 598 participants were initially selected with both genetic and phenotypic data, comprising 13 108 men and 20 490 women aged from 40 to 77. Among the 33 598 participants with genetic data, 25 406 participants were finally selected who had measures of serum bilirubin levels. Serum total bilirubin levels (both direct and indirect bilirubin) were measured through automated biochemical profiling, and the unit of bilirubin concentration was milligram per deciliter. General characteristics of KoGES population are shown in Table I in the online-only Data Supplement and further details concerning the KoGES can be obtained from the previously published cohort profile.^13^

Genetic data were obtained using the Korea Biobank Array (referred to as Korean Chip) available through the K-CHIP consortium. Korean Chip, containing about 830 000 single nucleotide polymorphisms (SNPs) specific to the Korean population, was designed by the Center for Genome Science, Korea National Institute of Health, Korea (4845-301, 3000–3031).^14^ Standard quality control procedures (*P* value for Hardy-Weinberg equilibrium ≥1.0×10^−6^, call rate ≥95%, and INFO ≥0.8) were used. Genetic data were imputed using SHAPEIT v.2^15^ and Minimac v.3,^16^ utilizing the 1000 Genome reference panel phase 3, as well as 397 samples of Korean reference genome provided by the Center for Genome Science of Korea National Institute of Health.

We performed a genome-wide association study (GWAS) to identify SNPs associated with serum total bilirubin levels, adjusting for age and sex (minor allele frequency ≥0.01, *P* value for Hardy-Weinberg equilibrium test ≥0.0001). The GWAS of bilirubin was conducted without any transformation. The results are presented in Figure I in the online-only Data Supplement. Applying a Bonferroni multiple testing correction, a total of 1784 SNPs were identified to associated with serum total bilirubin levels at a genome-wide significance level (*P*<5×10^−8^). As 2-sample MR analyses generally require SNPs to be independent, we applied a linkage disequilibrium clumping algorithm to identify independent SNPs using an *R*^*2*^ threshold <0.005. After excluding correlated SNPs by clumping algorithm, a total of 10 SNPs were selected as genetic instruments for genetically determined serum bilirubin levels.

A replication of the bilirubin GWAS was also performed using the KCPS-II biobank (n=13 855), and the Human Gene Ontology names of the 10 SNPs were explored from NCBI or PhenoScanner database.^17^ Cross-referencing identified SNPs using online data resources such as PhenoScanner is valuable in evaluating agreement with previous GWAS analyses, as well as potentially highlighting associations which may violate the underlying MR assumptions. However, it should be acknowledged that as such resources are largely representative of Western populations, the extent to which they are applicable to non-Western populations must be taken into consideration.

Potential horizontal pleiotropic associations, violating assumptions IV2 and IV3 of the MR approach, were explored from the PhenoScanner database.^17^ We defined a SNP below GWAS significance (*P*<5×10^−8^, proxies for East Asian: *R*^2^>0.8) as being potentially pleiotropic, if the related phenotype had previously been reported as a cardiovascular risk factor.

### Data Sources for the Study Outcome

Total and ischemic stroke were selected as outcome variables, using data from the KCPS-II biobank. KCPS-II is comprised of 156 701 individuals who visited 18 health examination centers in the Seoul and Gyeonggi districts of Korea for a health check-up from 2004 to 2013. The participants were aged from 20 to 84, including 94 840 men and 61 861 women. Further details about KCPS-II biobank are presented in a previously reported cohort profile,^18^ and general characteristics of KCPS-II population are displayed in Table I in the online-only Data Supplement. As with the KoGES sample, genetic data were produced using Korean Chip, using identical quality control and imputation methods.

Total stroke was defined as all forms of stroke including ischemic, hemorrhagic, and all other stroke subtypes, including both prevalent and incident cases of stroke. *International Classification of Diseases-Tenth* Revision codes were used to define stroke and stroke subtype cases, specifically: ischemic stroke, I63–I639; hemorrhagic stroke, I60–I629; and all stroke types, I60–I699.

Stroke cases were determined using hospital admission discharge records from 2005 to 2015 (median follow-up duration, 8.0 years), available from health insurance claims provided by the National Health Insurance Service.^10^ A total of 1489 total stroke cases were identified (654 ischemic stroke, 340 hemorrhagic stroke, 32 ischemic-hemorrhagic combined stroke, and 463 all other stroke subtypes), and 686 ischemic stroke (654 ischemic stroke and 32 ischemic-hemorrhagic combined stroke). To conduct a GWAS of stroke risk, 12 366 controls were used, fitting a logistic regression model adjusting for age and sex (minor allele frequency ≥0.01, *P* value for Hardy-Weinberg equilibrium test ≥0.0001). Log odds for stroke risk were then used in subsequent MR analyses.

Signed written consent was obtained from all participants, and The Institutional Review Board of Yonsei University approved this study protocol (IRB approval number 4-2014-1008).

### Statistical Analysis

For each individual genetic instrument, an MR estimate can be obtained by dividing the instrument-outcome association (KCPS-II) by the instrument-exposure association (KoGES). This is referred to as a Wald ratio estimate, using association estimates obtained from GWASs. In this study, we specifically consider inverse variance weighted (IVW), MR-Egger, and weighted median regression approaches, which utilize the set of Wald ratio estimates provide more accurate estimates of causal effect, as well as test and correct for bias due to horizontal pleiotropic pathways.

The IVW estimate is obtained by regressing the set of instrument-outcome associations upon the instrument-exposure associations omitting an intercept and weighting each estimate by the inverse of the variance of the instrument-outcome association. This represents a weighted average of Wald ratio estimates. In the case of MR-Egger regression, an intercept is included, interpreting the intercept as an average horizontal pleiotropic effect across the set of genetic instruments and the slope of the regression as the corrected causal effect.^19^

It should be noted that as an additional parameter is estimated, MR-Egger is statistically less powerful than IVW. MR-Egger regression is also reliant on the magnitude of pleiotropic effects being independent of the strength of instrument-exposure association. Finally, weighted median regression essentially calculates a weighted median using the Wald ratio estimates, with the weighting corresponding to the inverse variance weights selected. This has the advantage of being robust to bias due to horizontal pleiotropic bias, provided the proportion of nonpleiotropic SNPs exceeds 50% with respect to their corresponding weighting.

Genetic instruments are required to be strongly associated with the exposure of interest (IV1), with the strength of association quantified using the *F*-statistic for the genetic instrument in a regression of the exposure on the instrument. We calculate *F*-statistics adopting the method put forward by Au Yeung et al.^20^ The mean *F*-statistics for the 10 genetic instruments was 168, satisfying the threshold of *F*>10, typically recommended for MR analyses.^21^ For reference, an *F*-statistic of 10 represents a relative bias of 10% toward the null in a 2-sample MR setting.

Initially, Wald ratio estimates using each of the 10 genetic instruments were constructed by dividing the log odds of stroke risk by the β coefficient of bilirubin levels, obtained from GWASs of KCPS-II and KoGES. With this complete, IVW, MR-Egger, and weighted median regression models were applied, interpreting the estimated association as the effect of a genetically determined 1 mg/dL increase in serum bilirubin levels on the stroke risk.^22^ Additionally, we performed leave-one-out analyses by systematically removing each SNP in turn and repeating the IVW analysis to assess if a single SNP is driving the association.^23^ For the MR-Egger regression, a significant nonzero intercept is considered evidence of horizontal pleiotropic bias (*P* value for MR-Egger intercept <0.05), and where this is not the case, the IVW estimate is preferable owing to the statistical power limitations of MR-Egger regression.

To improve the visualization of the IVW and MR-Egger estimates, we performed radial variants of the IVW and MR-Egger models. These models are similar to the conventional IVW and MR-Egger regression models, but regress the product of the Wald ratio estimate and the square root of the weighting for each genetic variant upon the square root of the genetic variants weighting. For radial IVW, an intercept is omitted in contrast to radial MR-Egger, as in the conventional setting. This has the advantage that genetic instruments which are subject to pleiotropic bias can be identified as outliers, based on their distance from the regression line. The distance of each point to the regression line is proportional to the individual contribution to global heterogeneity in effect estimates for the given genetic instrument.^24^

All MR analyses were calculated using the TwoSampleMR^25^ and RadialMR^24^ R packages, using R, version 3.5.1 (R Development Core Team, Vienna, Austria).

## Results

### Genetic Instrumental Variables for Bilirubin

The associations for each genetic instrument with serum bilirubin levels and stroke risk are presented in Table 1, and all genetic instruments were replicated within KCPS-II biobank participants (Table II in the online-only Data Supplement). Of the 10 genetic instruments, only rs2119503 had a readily identifiable pleiotropic association, having previously been reported to be associated with Crohn disease in European populations (*P*=1.94×10^−12^). A proxy variant for rs2119503, rs6758317,^26^ was also associated with Crohn disease in East Asian populations (*P*=4.50×10^−21^).^27^ Although all instrumental variables were located on the chromosome 2 or 12, there was no overlap in terms of overarching gene. The associations of stroke risk for each genetic variant are shown in Table 1 on the log odds scale.

**Table 1.**
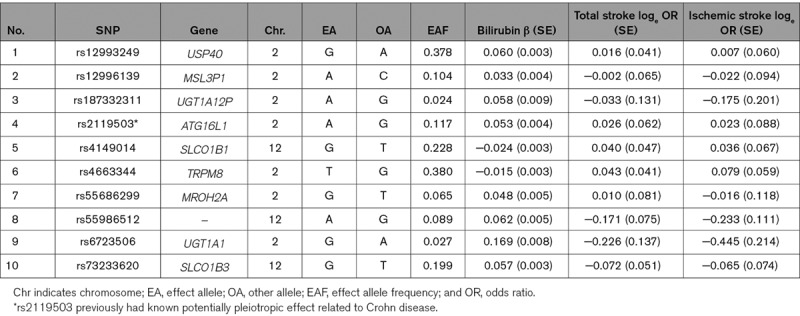
List of Genetic Instruments for Serum Bilirubin Levels and Log Odds Ratios of Stroke Risk by Each Instrumental SNPs (GWAS Significance with *P*<5×10^−8^ and linkage disequilibrium threshold with *R*^2^<0.005)

### Mendelian Randomization Analyses for Stroke Risk

**Figure 1.**
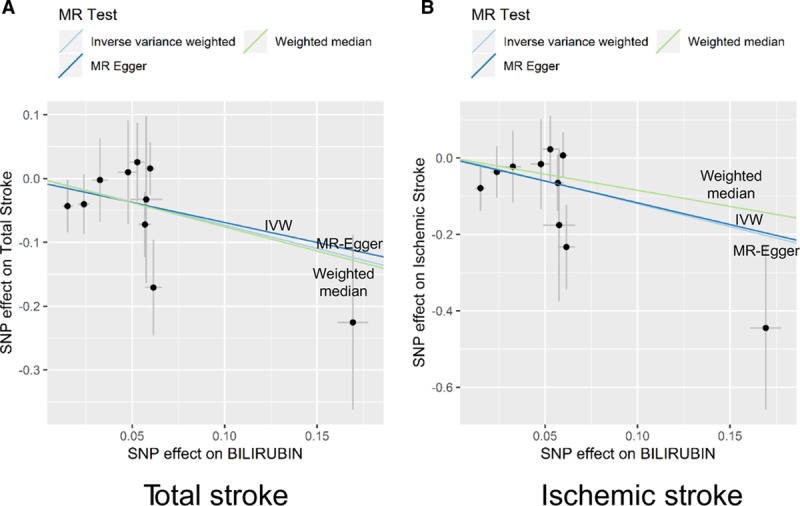
**Scatter plot to visualize causal effect of serum bilirubin on total stroke risk (A) and ischemic stroke risk (B).** The slope of the straight line indicates the magnitude of the causal association. IVW indicates inverse-variance weighted; and MR, Mendelian randomization.

IVW, MR-Egger, and weighted median regression were used to estimate causal associations between genetically predicted serum bilirubin levels and stroke risk (Table 2; Figure 1). Using IVW, we found evidence of causal relationships between bilirubin levels and stroke risk. Serum bilirubin levels seem to be negatively associated with both total and ischemic stroke risk (total stroke: IVW odds ratio [OR] per 1 mg/dL increase in serum bilirubin=0.481 [95% CI, 0.234–0.988]; *P*=0.046; Ischemic stroke: IVW OR per 1 mg/dL increase in serum bilirubin=0.302 [95% CI, 0.105–0.868]; *P*=0.026). MR-Egger and weighted median regression also showed directionally similar estimates, though they seem insufficiently powered to exceed conventional significance thresholds (total stroke: MR-Egger OR per 1 mg/dL increase in serum bilirubin, 0.534 [95% CI, 0.125–2.286]; *P*=0.422; weighted median OR per 1 mg/dL increase in serum bilirubin, 0.469 [95% CI, 0.174–1.262]; *P*=0.134; ischemic stroke: MR-Egger OR per 1 mg/dL increase in serum bilirubin, 0.320 [95% CI, 0.037–2.781]; *P*=0.332; weighted median OR per 1 mg/dL increase in serum bilirubin, 0.430 [95% CI, 0.102–1.821]; *P*=0.252).

**Table 2.**
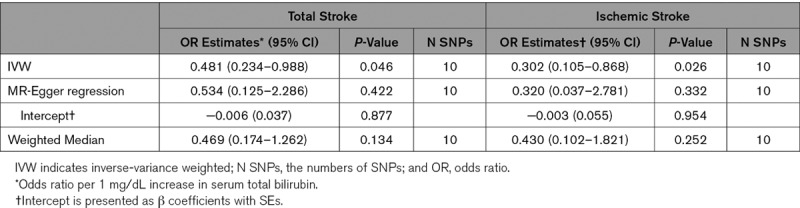
Mendelian Randomization for Serum Bilirubin on Stroke Risk

**Figure 2.**
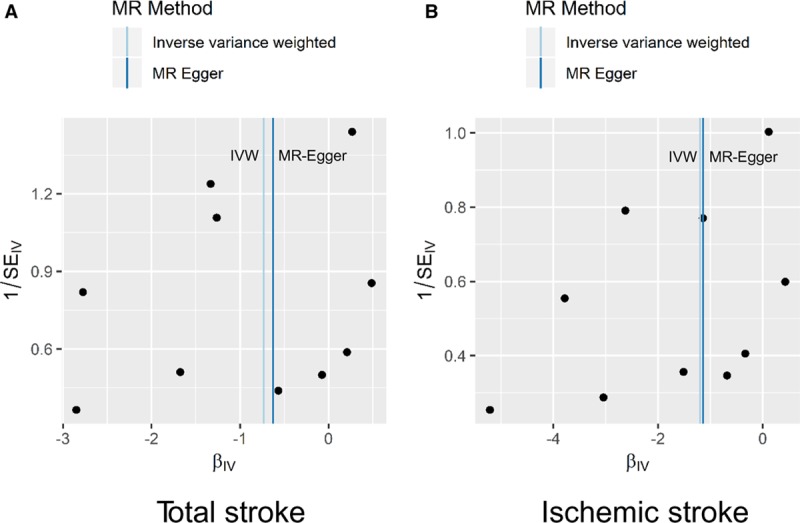
**Funnel plots to visualize overall heterogeneity of Mendelian randomization (MR) estimates for the effect of bilirubin on the total stroke (A) or ischemic stroke risk (B).** IVW indicates inverse-variance weighted.

Funnel plots display the individual Wald ratios for each SNP plotted against their precision, where asymmetry is indicative of directional horizontal pleiotropy. It should be noted, however, that assessing funnel plots with respect to symmetry is difficult when using a small number of genetic instruments (Figure 2). The MR-Egger intercepts show no evidence for significant directional pleiotropy, for both total and ischemic stroke (*P*=0.877 and *P*=0.954, respectively; Table 2). These results suggest that directional pleiotropic effects are not present between bilirubin levels and either total or ischemic stroke risk.

### Effects of Individual Genetic Instruments in Relation to Stroke Risk

To compare the influence of individual SNPs on the overall causal estimate, we performed leave-one-out analyses. When we systematically removed each SNP and repeated the MR analyses, there did not appear to be a substantial difference in estimated causal effect. It is therefore likely that estimated effects are not the result of a single genetic instrument (Figure II in the online-only Data Supplement).

**Figure 3.**
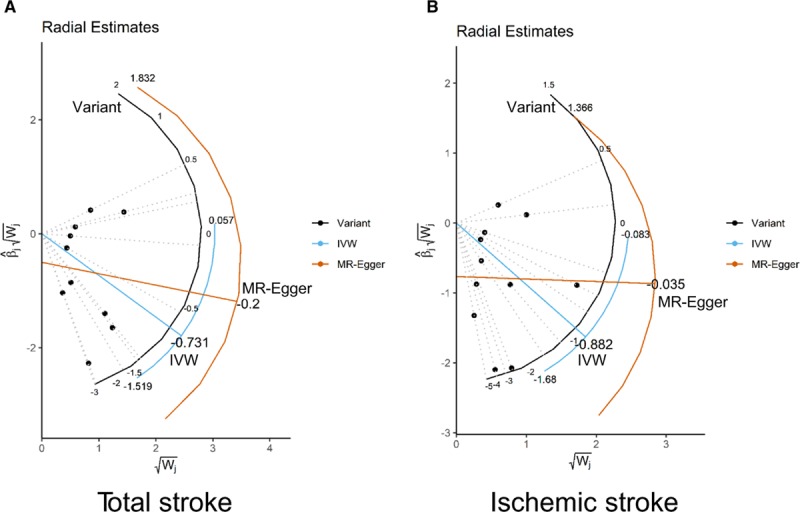
**Radial plots to visualize individual outlier single nucleotide polymorphisms (SNPs) in the Mendelian randomization (MR) estimates for total stroke (A) or ischemic stroke risk (B).** Black dots show valid SNPs and green dots display invalid outlier SNPs. There is no significant outlier SNP in these present plots. IVW indicates inverse-variance weighted.

When using radial IVW and MR-Egger approaches, there did not appear to be evidence of outlying genetic variants. This result is in agreement with the leave-one-out and MR-Egger analyses, which provide no indication of pleiotropic effects which could bias the overall analyses (Figure 3).

## Discussion

In this present study, we revealed that genetically determined serum total bilirubin levels are causally associated with decreased total stroke risk in a Korean population. This association increased in magnitude when restricting the analysis to ischemic stroke cases.

A number of observational studies in humans have shown evidence of strong associations between serum bilirubin levels and cardiovascular diseases. For example, serum total bilirubin concentrations were negatively correlated to arterial stiffness in Chinese men.^28^ Likewise, serum bilirubin levels were observed to be inversely associated with coronary artery calcification and cardiovascular events in a German population.^29^ In Chinese populations, serum total bilirubin levels were negatively associated with silent cerebral infarction, which increases the risk of transient ischemia attack, symptomatic stroke, and cardiovascular diseases.^30^ Regarding stroke risk, one National Health and Nutrition Examination Survey 1999 to 2004 study in the United States reported that serum total bilirubin levels were inversely associated with prevalent stroke and adverse stroke outcomes.^9^ In the Korean population specifically, serum bilirubin concentrations were estimated to be negatively associated with ischemic stroke in men.^10^

Using 2-sample summary MR, we investigated the causal relationship between circulating serum bilirubin levels and stroke risk. Interestingly, we found that genetically increased bilirubin levels are causally associated with decreased total stroke risk. When we limited the outcome to ischemic stroke, the magnitude of relevance became stronger. These results are consistent with previous observational studies and support the argument that previous MR studies lacked statistical power to detect such associations. As previous MR studies showed evidence of the inverse association, albeit with *P* values exceeding conventional thresholds, we strongly support the argument put forward by Davey Smith and Sterne,^31^ that findings should be interpreted with respect to the context of the study, and not be reduced to arbitrary thresholds.

Several experimental studies also support our findings. Vogel et al^32^ have revealed that bilirubin prevents atherosclerotic lesion formation in low-density lipoprotein receptor-deficient mice (*Ldlr*^−/−^) by inhibiting endothelial VCAM-1 (vascular cell adhesion molecule 1) and ICAM-1 (intercellular adhesion molecule 1)-mediated leukocyte migration. During atherosclerotic pathophysiology, binding of leukocyte integrins to endothelial VCAM-1 and ICAM-1 generates cellular reactive oxygen species production. These reactive oxygen species lead to alterations in the endothelial junctional structure that facilitate the transmigration of leukocytes. But bilirubin, a potent antioxidant, can remove the reactive oxygen species induced by the VCAM-1 and ICAM-1 activation that finally inhibit a migration of leukocyte and atherosclerotic lesion formation. This study group also has reported that bilirubin suppresses the transendothelial migration of murine lymphocytes in vitro^33^ and also attenuates tissue injury in mouse models of VCAM-1-dependent inflammation.^34^ Another study group has also suggested that bilirubin has antiproliferative effects on vascular smooth muscle cells in vitro through p53-dependent cell cycle arrest by hypophosphorylation of the retinoblastoma tumor suppressor protein in growth factor–stimulated vascular smooth muscle cells.^35^ Collectively, the putative biological mechanisms linking serum bilirubin to stroke risk are bilirubin-mediated inhibition of lipid oxidation, immune cell migration, and vascular cell proliferation.^36^

The present 2-sample summary MR has several strengths compared with one-sample MR. First, in 2-sample MR design, exposure and outcome variables are extracted from 2 independent populations, respectively. This is advantageous in cases where exposure and outcome data are not available in the same set of individuals.^37^ In this study, serum bilirubin levels were measured in KoGES, but stroke cases were not followed in this population. However, using stroke cases in the KCPS-II biobank, a homogenous population in terms of ethnicity and demographic profile, MR analyses could be performed. Further, utilizing multiple samples improves the overall sample size and subsequent precision of causal effect estimates. As a consequence, we were able identify a causal association between bilirubin levels and decreased stroke risk where our previous one-sample MR study was unable.^12^ The 2-sample summary MR approach is also useful in not restricting researchers to using individual level data. In this study, summary estimates were extracted from available individual level data; however, the methods can be applied in principle using publicly available GWAS data, as demonstrated by the MR-Base online platform.^38^

Two sample summary MR also has several characteristics which improve upon analyses using a single sample. In cases where the instrument is not strong, violating the first MR assumption (IV1), effect estimates will be attenuated toward the null. This provides a more conservative estimate, as opposed to the one-sample setting in which weak instruments result in bias toward the observational estimate. A related advantage with respect to the underlying MR assumptions is that the 2-sample study design is ideal for sensitivity analyses such as MR-Egger regression. As such, in performing a 2-sample summary MR, it is possible to perform a greater range of sensitivity analyses and arrive at more robust estimates of causal effect.

There are several limitations to this study. First, due to using summary data, any potential nonlinear relationships or stratification effects cannot be explored. Several previous epidemiological studies have argued that the association between bilirubin and cardiovascular risk resembles a *U*-shape^39,40^ and differs by sex.^10,28,40^ However, our previous work using quintile grouping by bilirubin level showed inversely linear associations between bilirubin and ischemic stroke risk in men, whereas all stroke cases showed no evidence of association. This result suggests that the observed *U*-shape association could potentially be driven by epidemiological studies failing to account for subtypes of CVD.^10^ Second, the absence of horizontal pleiotropic pathways is difficult to assess and if violated would result in biased estimates using IVW regression. We used MR-Egger and weighted median approaches to test for pleiotropic effects; however, each approach relies on a set of assumptions which are, at present untestable. Pleiotropic could be present where a majority of genetic instruments exhibit similar bias or where the instrument strength is associated with the magnitude of pleiotropic effect. The absence of outliers supports these assumptions holding, though the possibility cannot be discounted.

As a third limitation, the clumping algorithm used to identify correlated genetic variants relied primarily on European samples from the 1000 genomes project. This is owing to the lack of corresponding data for East Asian populations. However, as none of the genetic instruments shared a common gene, it seems probable that the conventional clumping algorithm is sufficient in this case. This is further supported by the inclusion of SNPs located on the *UGT1A1* and *SLCO1B1* genes, which encode essential proteins for the bilirubin metabolism. This shows that the genetic variants selected remain biologically plausible. Finally, it is important to emphasize that as this study focuses solely upon effects within a Korean population, the results cannot be immediately generalized to other ethnicities without further justification.

## Conclusions

In summary, we have shown evidence of causal relationships between circulating serum bilirubin levels and decreased stroke risk in Korean population. These results support the conclusions of previous observational studies between bilirubin and oxidative stress-related diseases such as stroke and atherosclerosis. This study therefore contributes to a growing body of evidence suggesting bilirubin could play a role as a therapeutic target to cardiovascular diseases.

## Acknowledgments

We thank the staff and participants from the Health Insurance Policy Research Institute of National Health Insurance Service for their valuable contributions. This study was conducted with bioresources from National Biobank of Korea, the Center for Disease Control and Prevention, Republic of Korea (KBN-2016-013).

## Sources of Funding

This study was funded by a grant from the Korean Health Technology R&D Project, Ministry of Health & Welfare, Republic of Korea (HI14C2686).

## Disclosures

None.

## Supplementary Material
