# Supplementary material for: Causal Associations Between Serum Bilirubin Levels and Decreased Stroke Risk: A Two-Sample Mendelian Randomization Study
Source: Arterioscler Thromb Vasc Biol. 2019 Dec 5;40(2):437–45. doi: 10.1161/ATVBAHA.119.313055 (PMC6975519; doi:10.1161/ATVBAHA.119.313055)
Supplement: Supplementary file 1 [file atv-40-437-s001.pdf]

SUPPLEMENTAL MATERIAL

Causal associations between serum bilirubin levels and decreased stroke risk : A two-sample Mendelian randomization study

Yoonjeong Choi, MSc [1,2,†], Sun Ju Lee, PhD [2,4,†], Wes Spiller, MA, MSc [3], Keum Ji Jung, PhD [2], Ji-Young Lee, PhD [2], Heejin Kimm, MD, PhD [2], Joung Hwan Back, PhD [4], Sunmi Lee, PhD [4], Sun Ha Jee, PhD [1,2‡]

Contents: Supplemental table 2, Supplementary figure 2

[1] Department of Public Health, Graduate School, Yonsei University, Seoul 03722, Korea

[2] Department of Epidemiology, Institute for Health Promotion, Graduate School of Public Health, Yonsei University, Seoul 03722, Korea

[3] Population Health Science Institute, University of Bristol, Barley House, Oakfield Grove, Bristol, BS8 2BN, U.K

[4] Health Insurance Policy Research Institute, National Health Insurance Service, Wonju, Korea

[†] Yoonjeong Choi and Sun Ju Lee contributed equally to this work.

[‡] Sun Ha Jee, PhD, MPH (Corresponding Author):

Department of Epidemiology and Health Promotion, Graduate School of Public Health,

Yonsei University, 50-1 Yonsei-ro, Seodaemun-gu, Seoul 03722, Republic of Korea

Tel: 82-2-2228-1523; E-mail: jsunha@yuhs.ac; Fax: 82-2-392-7734

Supplementary table Ⅰ. General characteristics of study populations

	KoGES (n=25,406)	KCPS-II (n=13,855)
Age, years	53.29 (8.33)	45.90 (10.91)
BMI, kg/m^2	24.15 (2.98)	23.97 (3.09)
Systolic blood pressure, mmHg	122.41 (15.90)	120.38 (14.98)
Fasting blood glucose, mg/dL	93.75 (20.55)	94.31 (21.75)
Total cholesterol, mg/dL	197.31 (35.82)	191.60 (34.44)
LDL-cholesterol, mg/dL	118.88 (32.25)	114.47 (32.14)
Triglyceride, mg/dL	135.40 (96.40)	142.59 (94.77)
Total bilirubin, mg/dL	0.71 (0.31)	-
Min, mg/dL	0.11	
25th percentile, mg/dL	0.50	
50th percentile, mg/dL	0.70	
75th pertcentile, mg/dL	0.85	
95th percentile, mg/dL	1.30	
Max, mg/dL	8.67	
Total stroke incidence, n	-	1,489
Ischemic stroke incidence, n	-	654
Hemorrhagic stroke, n	-	340
Both ischemic and hemorrhagic stroke, n	-	32
Other stroke subtypes, n	-	463

Data are expressed as mean (SD) unless otherwise indicated.

Supplementary table Ⅱ. Replication of the bilirubin GWAS in KCPS-II biobank (n=13,855)

No.	SNP	Gene	Chr.	EA	Bilirubin β (S.E.)	P-value
1	rs12993249	*USP40*	2	G	0.066 (0.004)	1.78E-49
2	rs12996139	*MSL3P1*	2	A	0.032 (0.007)	5.76E-06
3	rs187332311	*UGT1A12P*	2	A	0.062 (0.014)	1.63E-05
4	rs2119503	*ATG16L1*	2	A	0.048 (0.007)	1.11E-12
5	rs4149014	*SLCO1B1*	12	G	– 0.030 (0.005)	5.19E-09
6	rs4663344	*TRPM8*	2	T	– 0.023 (0.004)	2.83E-07
7	rs55686299	*MROH2A*	2	G	0.048 (0.009)	5.76E-08
8	rs55986512	-	12	A	0.074 (0.008)	3.94E-22
9	rs6723506	*UGT1A1*	2	G	0.229 (0.014)	9.16E-62
10	rs73233620	*SLCO1B3*	12	G	0.073 (0.005)	7.86E-41

Abbreviations: Chr, chromosome; EA, effect allele; OA, other allele; S.E, standard error.

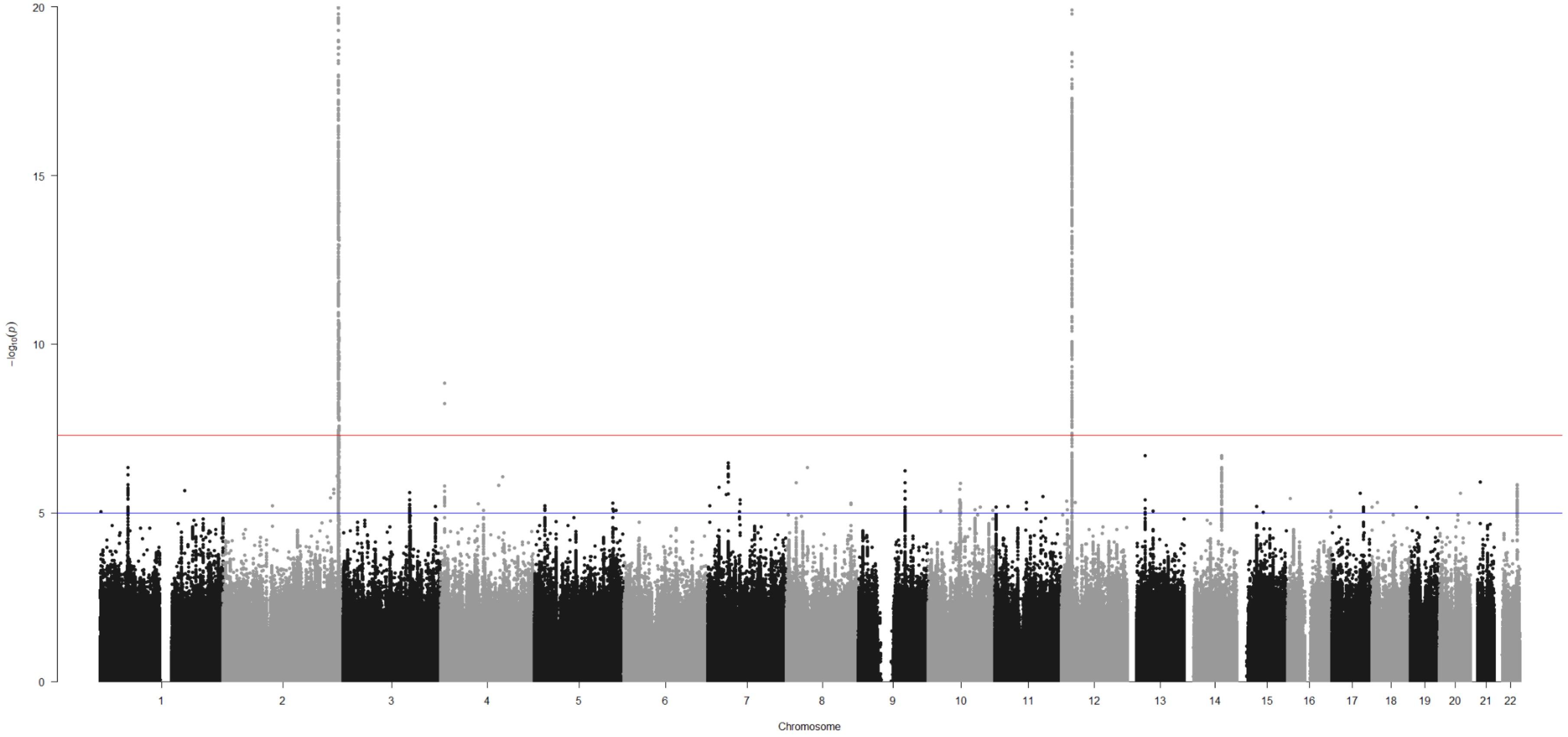

Supplemental figure Ⅰ. Manhattan plot of bilirubin GWAS (KoGES, n=25,406)

A

B

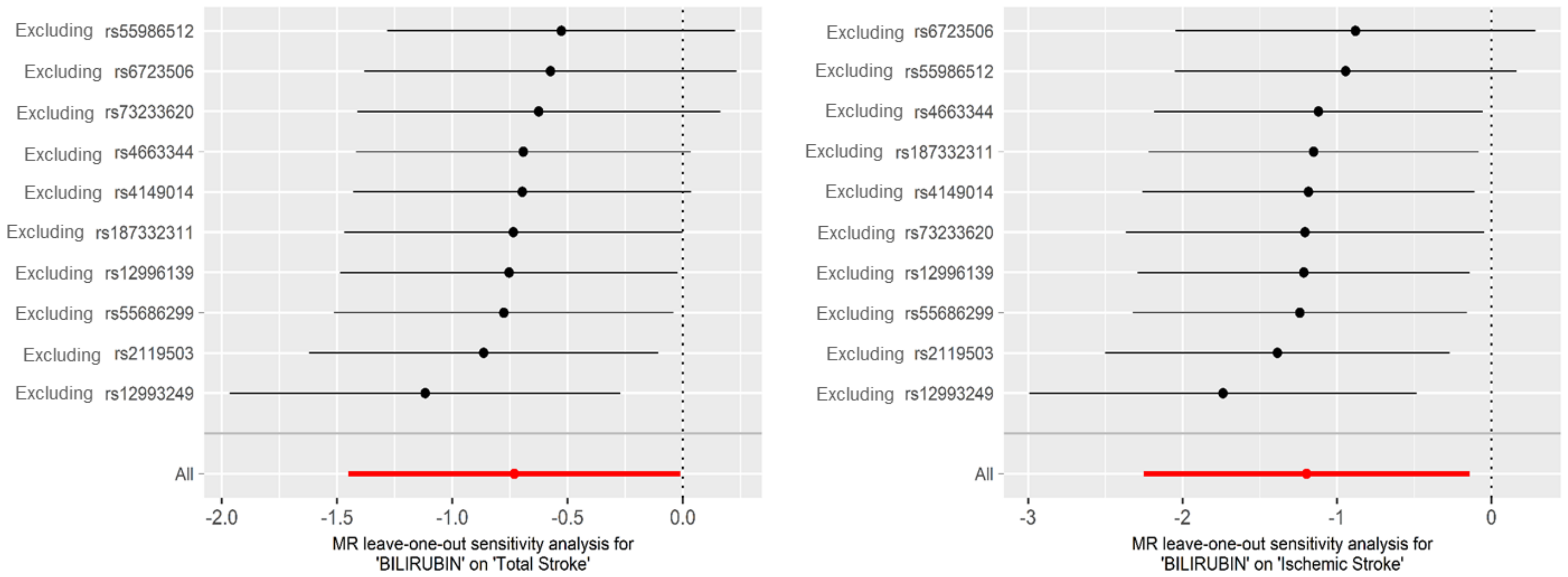

Supplemental figureⅡ. Leave-one-out sensitivity analysis of total stroke (A) and ischemic stroke (B) to investigate the possibility of causal association driven by a particular SNP.

Major Resources Tables

Animals (in vivo studies)

Species	Vendor or Source	Background Strain	Sex
NA	NA	NA	NA

Animal breeding

	Species	Vendor or Source	Background Strain	Other Information
**Parent - Male**	NA	NA	NA	NA
**Parent - Female**	NA	NA	NA	NA

Antibodies

Target antigen	Vendor or Source	Catalog #	Working concentration	Lot # (preferred but not required)
NA	NA	NA	NA	NA

Cultured Cells

Name	Vendor or Source	Sex (F, M, or unknown)
NA	NA	NA
