# Supplementary figures and images for: Causal Associations Between Serum Bilirubin Levels and Decreased Stroke Risk: A Two-Sample Mendelian Randomization Study
Source: Arterioscler Thromb Vasc Biol. 2019 Dec 5;40(2):437–45. doi: 10.1161/ATVBAHA.119.313055 (PMC6975519; doi:10.1161/ATVBAHA.119.313055)

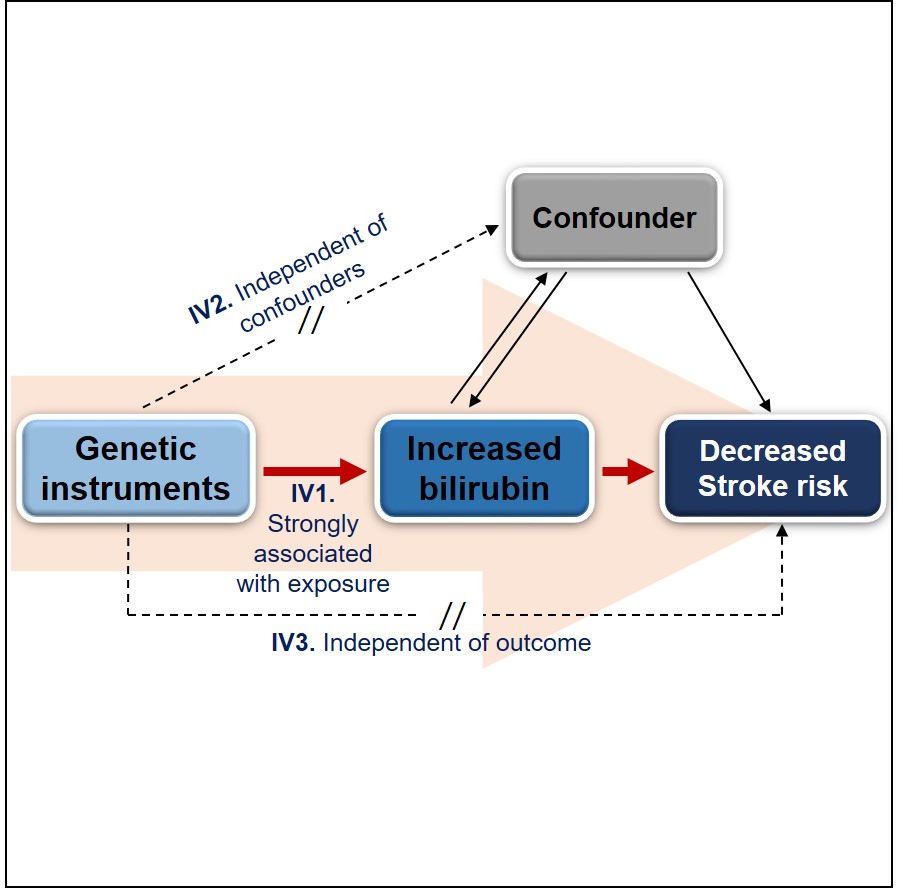

Supplement: Supplementary file 2 [file atv-40-437-s002.jpg]
